# Differentiating *Entamoeba histolytica*, *Entamoeba dispar* and *Entamoeba moshkovskii* using nested polymerase chain reaction (PCR) in rural communities in Malaysia

**DOI:** 10.1186/1756-3305-5-187

**Published:** 2012-09-04

**Authors:** Romano Ngui, Lorainne Angal, Siti Aminah Fakhrurrazi, Yvonne Lim Ai Lian, Lau Yee Ling, Jamaiah Ibrahim, Rohela Mahmud

**Affiliations:** 1 Department of Parasitology, Faculty of Medicine, University of Malaya, 50603, Kuala Lumpur, Malaysia

## Abstract

**Background:**

In this study, a total of 426 human faecal samples were examined for the presence of *Entamoeba histolytica, Entamoeba dispar, Entamoeba moshkovskii* infection via a combination of microscopic examination and nested polymerase chain reaction (PCR) targeting 16S ribosomal RNA of *Entamoeba* species.

**Methods:**

Faecal sample were collected from 426 participants in five rural villages in Peninsular Malaysia. The faecal samples were processed by direct wet smear and formalin ethyl acetate concentration technique followed by iodine staining and examined via microscopy for the presence of *Entamoeba* species and other intestinal parasites. Microscopically positive samples for *Entamoeba* species cysts were further characterized using a Nested Polymerase Chain Reaction (Nested-PCR) targeting 16S-like ribosomal RNA gene. The data entry and analysis was carried out using the SPSS software (Statistical Package for the Social Sciences) program for Windows version 17 (SPSS, Chicago, IL, USA).

**Results:**

Based on single faecal examination, overall prevalence of *Entamoeba* infection was 17.6% (75/426). Females (19.1%) were more commonly infected compared to males (15.9%). Comparison by age groups showed that adults (23.9%) had higher infection rates than children (15.3%). The PCR results showed that 52 out of 75 microscopy positive samples successfully generated species-specific amplicons. The infection with *E. histolytica* (75.0%; 39/52) was the most common, followed by *E. dispar* (30.8%; 18/52) and *E. moshkovskii* (5.8%; 3/52). Of these, 33 (63.5%) were shown to contain only *E. histolytica,* 10 (19.2%) contained *E. dispar* and 3 (5.8%) contained only *E. moshkovskii*. Mixed infection with *E. histolytica* and *E. dispar* was found in 6 (11.5%) samples.

**Conclusions:**

The present study essentially emphasized the benefit of molecular techniques in discriminating the pathogenic *Entamoeba* species from the non-pathogenic for accurate diagnosis and better management of amoebiasis. The presence of *E. moshkovskii* is of great public health concern as it was the first time it has been reported in Malaysia.

## Background

The genus * Entamoeba * comprises six species, namely * Entamoeba histolytica, E. dispar, E. moshkovskii, E. coli, E. hartmanni * and * E. polecki * that live in the human intestinal lumen. Infections with * Entamoeba * species can result in either a harmless colonization of the intestine or invasion of the colonic wall and damage of other host tissues such as liver, lung and brain. Most of the * Entamoeba * species are commensal parasites and do not cause human disease. Amoebiasis which is caused by * Entamoeba histolytica * is a global health problem as it is responsible for more than 100,000 deaths per year and is the second leading cause of global death due to protozoa after malaria [1-3]. Major symptoms of amoebiasis are abdominal pain, diarrhea, nausea, vomiting and flatulence. This infection is more preponderant in children compared to adults [4] and is commonly found in tropical and subtropical areas.

It has been reported that 10% of the world’s population are infected with * Entamoeba * species, in which pathogenic * E. histolytica * constitute 10% of these infections and the remaining 90% are infected by non-pathogenic * E. dispar *[5]*.* However, a recent study highlighted the existence of another species of *Entamoeba* known as * E. moshkovskii * which can also cause infection among humans [6]. * E. histolytica, E. dispar * and * E. moshkovskii * are morphologically identical but are different biochemically and genetically [6-8]. Although a previous study showed * E. moshkovskii * to be a non-pathogenic parasite, intestinal symptoms including diarrhea and other gastrointestinal disorders in individuals infected with this species have been reported [8-10]. This, however, has been rebutted by Al-Harthi and Jamjoon (2007) [11] who claimed that * E. moshkovskii * has never been associated with any disease. This may indicate that perhaps humans are a true host for this putatively free-living amoeba and are not just transiently infected. Therefore, the true prevalence of * E. dispar * and * E. moshkovskii * infections need to be investigated in order to determine their significant pathogenic potential in humans.

Traditionally, * Entamoeba * infections are diagnosed through microscopic examination of fresh or fixed faecal samples. However, very often * E. histolytica * cysts and trophozoites cannot be morphologically differentiated from * E. dispar* and * E. moshkovskii * through microscopic examination. Only when ingested red blood cells are present in trophozoites of * E. histolytica, * the ability to distinguish them from those of * E. dispar * and * E. moshkovskii * becomes easier. Recently, sensitive and specific serological and molecular techniques that are able to distinguish * E. histolytica * from * E. dispar * have been developed [10,12-20]. These include the detection of * E. histolytica * antigen using an enzyme-linked immunosorbent assay (ELISA) [16,17,19], the detection of * E. histolytica * by monoclonal antibodies of * E. histolytica * that specifically recognize * E. histolytica * antigen [12,15] and the use of the polymerase chain reaction (PCR) to amplify amoebic DNA [10,13,14]. More current approaches include the discrimination of *E. histolytica E. dispar * and * E. moshkovskii * by a simultaneous detection using multiplex nested PCR [18,20].

In Malaysia, intestinal parasitic infections (IPIs) including *Entamoeba* infections are more prevalent in rural areas especially among aboriginal communities compared to urban areas [21-23]. A recent study conducted by Ngui * et al *. (2011) among communities living in rural areas of Malaysia, showed that 10.2% of the participants were infected with * Entamoeba *. Another study showed that the infection rate of * Entamoeba * in rural communities in Malaysia was 21.0% [24]. Other local studies which include aboriginal groups reported prevalence ranging from 9.4% to 18.5% [25,26]. However, these prevalence rates were based on microscopic examination which could not differentiate between * E. histolytica  E. dispar * and * E.moshkovskii * infections.

In order to avoid unnecessary treatment of individuals with non-pathogenic * Entamoeba * species, it is important to discriminate these species from the pathogenic * E. histolytica *[1]. Additionally, there is also a need for simpler and better identification of these infections, not only for diagnostic purposes and care management, where * E. dispar * and * E. moshkovskii * infected individuals could be treated unnecessarily with antiamoebic chemotherapy, but also for a better understanding of the epidemiology of these parasites in the human population. Within this context, we conducted this study to determine the prevalence of *Entamoeba* species and the true * E. histolytica, E. dispar * and * E. moshkovskii * infections in human faecal samples using molecular techniques.

## Methods

### Study area and population

**Figure 1:**
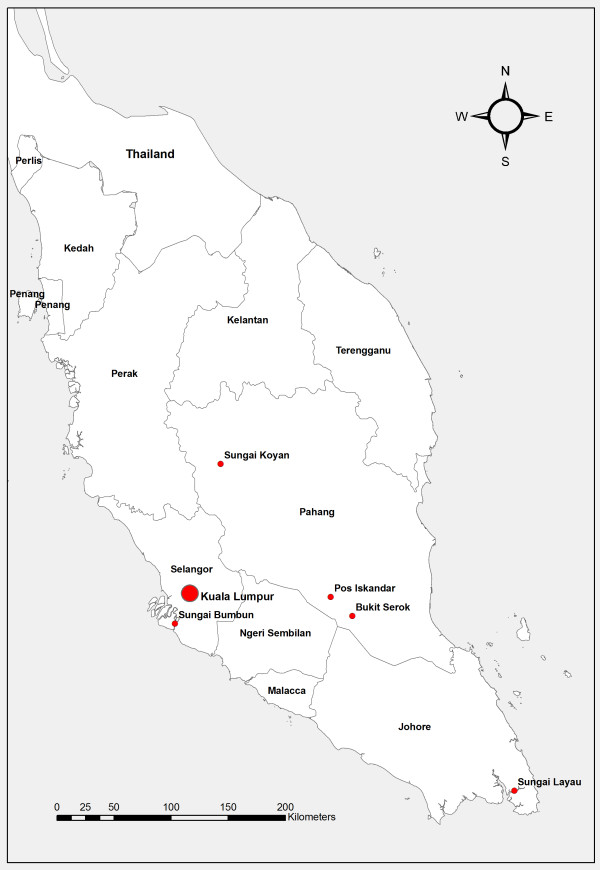
Location of the sampling areas.

The present study was carried out from 2009 to 2011 in five rural villages, namely Pos Iskandar (102.65°E longitude, 3.06°N latitude), Sungai Koyan (101.63°E longitude, 4.25°N latitude), Sungai Bumbun (101.42°E longitude, 2.85°N latitude), Bukit Serok (102.82°E longitude, 2.91°N latitude) and Sungai Layau (104.10°E longitude, 1.53°N latitude) (Figure 1). In brief, the villages are inhabited by members of the indigenous ethnic groups. Each village has a small population and the number of residents in each village is estimated to be 80 to 100 inhabitants. They live in deprived circumstances where overcrowding, poor environmental sanitation, low levels of education and poor provision of safe water are widespread. Although provisions of basic facilities are provided by local authorities, most of them could not afford to pay their monthly utility bills leading to the termination of water supplies. Therefore, rivers located adjacent to the village remain their main source of water for domestic needs such as drinking, cooking, bathing and washing clothes. The condition of the surrounding environment of the village is also generally poor with limited provision of latrine facilities therefore encouraging defecation in and around bushes or nearby rivers. Children usually defecate indiscriminately around their houses without parental supervision. Cats, dogs and poultry are the most common domestic animals. Most of these domestic animals are left to roam freely within the village and were observed to defecate in the surrounding property of their owners. The villagers have very close contact with these animals, even sharing food from the same plate with them.

### Consent, sample collection and laboratory procedures

The study protocol was approved by the Ethics Committee of the University of Malaya Medical Centre, Malaysia (UMMC; MEC Ref. No. 824.11). Before sample collection, an oral briefing to describe the objectives and methodology of the study was given to the participants by the investigator. Their consent was taken either in written form (signed) or verbally followed by thumb prints (for those who were illiterate) or their parents/guardians (on behalf of their children) after which pre-labeled plastic containers for faecal collection were handed out to all participants. Their ability to recognize their names was checked.

The filled containers were collected on the following day. The fresh faecal samples were stored at ambient temperature and transferred to the laboratory within 2–4 hours post-collection. Upon arrival at the laboratory, the samples were preserved in 2.5% potassium dichromate and kept at 4°C until further analysis. The faecal samples were processed by direct wet smear and formalin ethyl acetate sedimentation technique followed by iodine staining and examined via microscopy for the presence of *Entamoeba* species and other intestinal parasites*.* Microscopically positive samples for *Entamoeba* species cysts were further characterized using a molecular technique.

### Extraction of *entamoeba* genomic DNA

DNA was extracted from microscopically positive faecal samples using PowerSoil® DNA Isolation Kit (Mo Bio, cat. no. 12888–100, CA, USA) according to the manufacturer’s instructions. Briefly, approximately 0.2 to 0.3 g of faecal sample was added into the PowerBead Tube® followed by incubation at 70°C for 10 minutes with the presence of cell lysis and disruption agent provided in the kit. Afterwards, homogenization and cell lysis procedure of the faecal sample were completed by mechanical shaking (vortexing) using MO BIO Vortex Adapter (MO BIO, cat. no. 13000-V1). The extracted DNA was stored at −20°C until required for PCR amplification.

### Nested polymerase chain reaction (nested-PCR)

Nested PCR targeting 16S-like ribosomal RNA gene was used to genetically characterize *E. histolytica, E. dispar* and *E. moshkovskii* according to Que and Reed (1991) [27]. Primary PCR for the detection of *Entamoeba* genus used forward primer E-1 (5’-TAA GAT GCA GAG CGA AA-3’) and reverse primer E-2 (5’-GTA CAA AGG GCA GGG ACG TA-3’). Control samples without DNA (DNase free water, Sigma Cat. no. W4502) and with *Entamoeba* species genomic DNA (positive control) were included in each PCR run. The PCR was carried out in a 25 μl volume with the final mix containing 10× PCR buffer, 1.25 mM dNTPs, 25 mM MgCl_2_, 10 pmole of each primer, 2.5 U of *Taq* polymerase and 2.5 μl of DNA template. The sample was heated to 96°C for 2 min, followed by 30 cycles of 92°C for 1 min (denaturing), 56°C for 1 min (annealing), 72°C for 1 min 30 s (extension) and a final extension at 72°C for 7 min.

Subsequently, the primary PCR products were subjected to secondary PCR for *Entamoeba* species-specific characterization. Amplification was achieved using primer sets EH-1 (5’-AAG CAT TGT TTC TAG ATC TGA G-3’) and EH-2 (5’-AAG AGG TCT AAC CGA AAT TAG-3’) to detect *E. histolytica* (439 bp); ED-1 (5’-TCT AAT TTC GAT TAG AAC TCT-3’) and ED-2 (5’-TCC CTA CCTATT AGA CAT AGC-3’) to detect *E. dispar* (174 bp); Mos-1 (5’-GAA ACC AAG AGT TTC ACA AC-3’) and Mos-2 (5’-CAA TAT AAG GCT TGG ATG AT-3’) to detect *E. moshkovskii* (553 bp) [14] (Khairnar and Parija, 2007). The secondary amplification reagent concentrations were similar to the first PCR except that 2.5 μl of primary PCR product was added instead of genomic DNA template.

The secondary PCR had a similar cycling condition except that the annealing temperature (48°C instead of 56°C) and extension duration (1 min instead of 1 min 30 sec) were modified. In both amplifications, samples were incubated in the MyCycler thermal cycler (Bio-Rad, Hercules, USA). PCR generated amplicons of 174 bp for *E. dispar*, 439 bp for *E. histolytica* and 553 bp for *E. moshkovskii* were subjected to electrophoresis in 2% agarose gels at 100 V for 35 min and visualized in a UV transilluminator after staining with SYBR Safe DNA stain (Invitrogen, USA).

### Data analysis

Detection of *Entamoeba* species was determined on the basis of morphological characteristics of the cysts under microscopy. The average cyst size is 12 μm but ranges from 10–20 μm in diameter. The data entry and analysis was carried out using the SPSS software (Statistical Package for the Social Sciences) program for Windows version 17 (SPSS, Chicago, IL, USA). Qualitative data were estimated and presented as frequencies and percentage. The prevalence and 95% confidence intervals (CIs) were calculated for each parasite. Associations between proportions were explored using Chi-Square *X*^2^ (test) and a *P* value of <0.05 was considered indicative of a statistically significant difference.

## Results

### Prevalence of *Entamoeba* infection via microscopy

A total of 426 samples were collected and 75 (17.6%; 95% CI = 14.0-21.2%) samples were microscopically positive for *Entamoeba* cysts, either singly or in combination with other intestinal parasites (Table 1). The overall prevalence of other intestinal parasites has been published (Ngui *et al*. 2011). Infection was more prevalent in females (19.1%; 95% CI = 14.0-24.2%) compared to males (15.9%; 95% CI = 10.8-21.0%), however, it was not statistically significant (data not shown). Similarly, more adults (23.9%; 95% CI = 16.0-31.8%) were found to be positive for *Entamoeba* infections compared to children (15.3%; 95% CI = 11.3-19.3%), but this difference was also not significant (*P* > 0.05). Higher prevalence rates were recorded in Pos Iskandar (29.2%), followed by Sungai Koyan (22.2%), Sungai Bumbun (16.8%), Bukit Serok (15.3%) while Sungai Layau (5.6%) had the least (Table 2).

**Table 1:** **Overall prevalence of* Entamoeba *infection in the studied populations as determined by microscopy according to gender and age groups (N = 426)**

**Characteristics**	**N**	**n**	**%**	**95% CI**
Gender				
Male	201	32	15.9	10.8-21.0
Female	225	43	19.1	14.0-24.2
Age groups				
Children	313	48	15.3	11.3-19.3
Adults	113	27	23.9	16.0-31.8
**Total (Overall prevalence)**	**426**	**75**	**17.6**	**14.0-21.2**

N: Total number examined; n: * Entamoeba * positive by microscopy;%: Percentage; 95% CI: Confidence Interval.

**Table 2:** **Prevalence of* Entamoeba *infection based on microscopy and nested PCR assay of human faecal samples according to locations**

**Location (s)**	**No.**	**Microscopy**		**PCR assay**	
	**Examined**	**n**	**%**	**n**	*%
Pos Iskandar	113	33	29.2	29	87.9
Sungai Koyan	18	4	22.2	4	100
Sungai Bumbun	107	18	16.8	8	44.4
Bukit Serok	99	15	15.3	7	46.7
Sungai Layau	89	5	5.6	4	80.0
**Total**	**426**	**75**	**17.6**	**52**	**69.3**

n: Number positive; * Based on the number positive by microscopy.

### Nested PCR

Of the 75 microscopy-positive samples, 52 (69.3%) samples were successfully amplified and characterized the *Entamoeba* species on the basis of its amplicons size using nested PCR. Additionally, 351 microscopy-negative samples were also examined using PCR and the results confirmed that these samples were negative for the *Entamoeba* infection. Every sample that was negative by PCR (23 samples) but positive by microscopy, was retested by PCR, and each was again found to be PCR negative. Of the 52 PCR positive results, *E. histolytica* infection (75.0%; 39/52) appeared to be the most predominant, followed by *E. dispar* (30.8%; 18/52) and *E. moshkovskii* (5.8%; 3/52). Of these, 33 (63.5%) were shown to contain only *E. histolytica,* 10 (19.2%) contained only *E. dispar* and 3 (5.8%) contained only *E. moshkovskii*. Mixed infection with *E. histolytica* and *E. dispar* was found in 6 (11.5%) samples (Table 3).

**Table 3:** **Prevalence of* E. histolytica, E. dispar *and* E. moshkovskii *infections as determined by nested PCR in microscopically positive faecal samples according to locations**

**Location (s)**	**PCR Positive**	*** E. histolytica *only**		*** E. dispar *only**		*** E. moshkovskii *only**		*** E. histolytica + E. dispar ***	
		**n**	**%**	**n**	**%**	**n**	**%**	**n**	**%**
Pos Iskandar	29	28	96.6	1	3.4	0	0	0	0
Sungai Koyan	4	0	0	2	50.0	0	0	2	50.0
Sungai Bumbun	8	0	0	5	62.5	1	12.5	2	25.0
Bukit Serok	7	2	28.6	1	14.3	2	28.6	2	28.6
Sungai Layau	4	3	75.0	1	25.0	0	0	0	0
**Total**	**52**	**33**	**63.5**	**10**	**19.2**	**3**	**5.8**	**6**	**11.5**

Pos Iskandar recorded the highest prevalence of *E. histolytica* (53.8%; 28/52), followed by Sungai Layau (5.8%; 3/52) and 2 (3.8%) samples each from Sungai Koyan, Sungai Bumbun and Bukit Serok. As for *E. dispar*, the highest prevalence was detected in Sungai Bumbun (13.5%; 7/52), followed by Sungai Koyan (7.8%; 4/52), Bukit Serok (5.8%; 3/52) and 2% each (1/52) for Pos Iskandar and Sungai Layau. Meanwhile, *E. moshkovskii* was only found in two villages with Bukit Serok (3.8%; 2/52) recorded as having the highest prevalence rate followed by one infection in Sungai Bumbun.

## Discussion

The present study reported an overall prevalence of *Entamoeba* species as determined by microscopy as 17.6% (75/426). In Malaysia, previous studies have also reported high prevalence of *Entamoeba* infection with prevalence rates ranging from 9.4% to 21.0% among rural communities [23-26,28]. In contrast, the most recent study among rural communities in Malaysia demonstrated 10.2% of the participants were infected with *Entamoeba* species [23]. The high prevalence of *Entamoeba* infection may be due to the transmission and pathogenesis as well as other risk factors which favor the persistence of this infection. Given its faecal-oral route, habits related to eating, defecation, personal hygiene, cleanliness and level of education may have an impact on the prevalence rates. The findings of this study confirmed a trend of high risk of infection with *Entamoeba* species among the rural population as shown by other local studies [21-23,25], where prevailing poverty, poor socioeconomic condition, low standards of sanitation and hygiene and lack of education attainment may contribute to high prevalence of *Entamoeba* infection.

Additionally, the use of untreated river water as a source for household needs could also lead to the increase in the transmission of *Entamoeba* infection. A study in Thailand conducted to determine the occurrence of *Entamoeba* species in water samples using molecular techniques has indicated that water is a possible source for transmission of *Entamoeba* to humans [29]. From our personal observation, the majority of the households have no toilet facilities. They often defecate indiscriminately in the bushes and nearby river at the back of their houses. These water sources may be highly polluted especially in rainy seasons, by rain runoff contaminated with cysts of parasites from human faeces. Another possible source of infection could also be from the consumption of water contaminated with *Entamoeba* cysts from faeces of infected wild or domestic animals as they may come to the river bank to drink and at the same time defecate in or near the river. This situation is further aggravated as the drinking of unboiled water is a common practice among this community. Therefore, these communities should be given health education and made aware of the danger of drinking unboiled or improperly boiled untreated water.

Our molecular technique showed that *E. histolytica* (75.0%) was found to be the most common species detected in this study, followed by *E. dispar* (30.8%) and *E. moshkovskii* (5.8%). Similarly, the only available species-specific study of *Entamoeba* species conducted in Malaysia found that *E. histolytica* (13.2%) was more prevalent compared to *E. dispar* (5.6%) [28]. Interestingly, the high prevalence of *E. histolytica* in the present study was in contrast to the worldwide distribution of *Entamoeba* species, which indicated that *E*. *dispar* is perhaps 10 times more common than *E*. *histolytica*[1,2,30], however, the local prevalence may vary significantly, thus necessitating the assessment of prevalence in different geographical regions. Similar observation also reported that 70.8% of patients were infected with *E. dispar*, compared to 4.5% of *E. histolytica* and 61.8% of *E. moshkovskii* in Australia [10]. A study in Brazil also showed that the prevalence of *E. dispar* (90%) was more frequent compared to *E. histolytica* (10%) among infected individuals [5]. A study in India also showed parallel findings, where 49.5% patients were infected with *E. dispar* and only 7.4% with *E. histolytica*[18]. Likewise, a study in the Netherlands also found 91.2% microscopic positive samples were identified as *E. dispar* while 6.7% were *E. histolytica* by PCR [31]. In Canada, 97.1% of the examined samples contained *E. dispar* compared to 2.9% of *E. histolytica* by both PCR and ELISA assay [16].

To the best of our knowledge, the detection *E. moshkovskii* (5.8%) in this study was the first to be reported in Malaysia. Cases of humans infected with *E. moshkovskii* have been reported sporadically from different parts of the world including Thailand [6], India [9,18,20], Bangladesh [8,32] and Australia [10]. A study in Bangladesh highlighted that infection with *E. moshkovskii* was common in children aged 2 to 5 years [8] while a study in India found that *E. moshkovskii* infection was associated with dysentery [9]. In our study, it was noted that all individuals infected with *E. moshkovskii* were children and were asymptomatic. Although amoebic liver abscess (65%) has been documented in patients admitted to an urban hospital in Malaysia [33], information from rural communities is not available as this infection can only be confirmed in a hospital setup. Therefore, future investigation which includes the clinical impact of *E. moshkovskii* and other *Entamoeba* species is imperative for a better understanding of a true pathogenic potential of *E. moshkovskii*.

Although every negative PCR sample was retested by PCR, each was again found to be negative. This result can potentially be explained by the presence of faecal inhibitor substances which were not completely eliminated prior to PCR reaction. Further study to optimize the reduction of these inhibitors is necessary during the extraction process in order to increase PCR sensitivity. It is also possible that the samples which were detected by microscopy but not PCR may belong to other *Entamoeba* species such as *E. coli, E. hartmanni* and *E. polecki*, or the contained a low number of parasites, which fell below the PCR detection limit. Therefore, a more sensitive method such as Real-Time PCR and employing primers for all *Entamoeba* species should be considered in future study. The failure to amplify samples could also be due to the fact that samples may contain only trophozoites that could have degenerated with time. Several studies have confirmed that the presence of *Entamoeba* cysts in the faecal samples, in contrast to trophozoites, somewhat increase the chances of the PCR assay [10,34].

## Conclusion

Molecular techniques are indeed promising tools for epidemiological studies, particularly in discriminating the pathogenic from the non-pathogenic species of the *Entamoeba* species. This study reports for the first time the identification of *E*. *moshkovskii* in human faecal samples from Malaysia. Since all of the infected participants with *E*. *moshkovskii* including those infected with the other two species were asymptomatic, further investigations are needed to determine the true pathogenic potential of these three species.

## Competing interests

The authors have declared that no competing interests exist.

## Authors’ contributions

RM, YALL, LYL and JI planned and designed the protocols. RN, LA and SAF conducted the field study the study programme, including the collections of stool samples and data from the questionnaire interviews, as well as the management of collected data. RM, YALL, LYL and JI supervised all the laboratory work. RN, LA and SAF carried out the data analysis and interpretation. RN, LA, YALL and RM prepared the first draft of the manuscript and all authors revised the manuscript critically. All authors read and approved the final version of the manuscript.

## Financial support

This research work was funded by the High Impact Research Grant (E00050-20001), University of Malaya Research Grant (RG221-10HTM) and Postgraduate Research Fund (PV024/2011B) from University of Malaya. The funders had no role in study design, data collection and analysis, decision to publish or preparation of the manuscript.

## References

[B1] WHO World Health Organization/Pan American Health Organization/UNESCO report of a consultation of experts on amoebiasis Wkly Epidemiol Rec WHO 1997 72 97 99

[B2] Markell EK John DJ Krotoski WA Lumen dwelling protozoa 1999 8 Philadelphia, USA: WB Saunders

[B3] Delialioglu N Aslan G Ozturk C Ozturhan H Sen S Emekdas G Detection of Entamoeba histolytica antigen in stool samples in Mersin, Turkey J Parasitol 2008 94 530 532 10.1645/GE-1355.1 18564756

[B4] Salvioli L Bundy DAP Tomkin A Intestinal parasitic infections Trans R Soc Trop Med Hyg 1992 86 353 354 10.1016/0035-9203(92)90215-X 1440799

[B5] Braga L Gomes ML Silva MW Paiva C Sales A Mann BJ Entamoeba histolytica and Entamoeba dispar infections as detected by monoclonal antibody in an urban slum in Fortaleza, Northeastern Brazil Rev Soc Bras Med Trop 2001 34 467 471 10.1590/S0037-86822001000500010 11600913

[B6] Hamzah Z Petmitr S Mungthin M Leelayoova S Chavalitshewinkoon-Petmitr P Differential detection of Entamoeba histolytica, Entamoeba dispar and Entamoeba moshkovskii by a single-round PCR Assay J Clin Microbiol 2006 44 3196 3200 10.1128/JCM.00778-06 16954247 PMC1594701

[B7] Clark CG Diamond LS The Laredo strain and other ‘Entamoeba histolytica like’ amoebae are Entamoeba moshkovskii Mol Biochem Parasitol 1991 46 11 18 10.1016/0166-6851(91)90194-B 1677159

[B8] Ali IKM Hossain MB Roy S Ayeh-Kumi P Petri JWA Haque R Clark CG Entamoeba moshkovskii infections in children, Bangladesh Emerg Infect Dis Emerging 2003 9 580 584 10.3201/eid0905.020548 PMC2972761 12737742

[B9] Parija SC Khairnar K Entamoeba moshkovskii and Entamoeba dispar-associated infections in Pondicherry, India J Health Popul Nutr 2005 23 292 295 16262027

[B10] Fotedar R Stark D Beebe N Marriot D Ellis J Harkness J PCR detection of Entamoeba histolytica, Entamoeba dispar and Entamoeba moshkovskii in stool samples from Sydney, Australia J Clin Microbiol 2007 45 1035 1037 10.1128/JCM.02144-06 17229864 PMC1829108

[B11] Al-Harthi SA Jamjoon MB Diagnosis and differentiation of Entamoeba infection in Makkah Al Mukarramah using microscopy and stool antigen detection kits World J Med Sci 2007 2 15 20

[B12] Wonsit R Thammapalerd N Tharavanji S Radomyos P Bunnarg D Enzyme-linked immunorsorbent assay based on monoclonal and polyclonal antibodies for the detection of Entamoeba histolytica antigens in faecal specimens Trans Roy Soc Trop Med Hyg 1992 86 166 169 10.1016/0035-9203(92)90553-O 1440778

[B13] Katzwinkel-Wladarsch S Loscher T Reider H Direct amplification and differentiation of pathogenic and non-pathogenic Entamoeba histolytica DNA from stool specimen Am Soc Trop Med Hyg 1994 51 115 118 10.4269/ajtmh.1994.51.115 8059910

[B14] Troll H Marti H Weiss N Simple differential detection of Entamoeba histolytica and Entamoeba dispar in fresh stool specimens by sodium acetate-acetic acid-formalin concentration and PCR J Clin Microbiol 1997 35 1701 1705 9196177 10.1128/jcm.35.7.1701-1705.1997 PMC229825

[B15] Yvonne CW Crandal I Kain CK Development of monoclonal antibodies which specifically recognize Entamoeba histolytica in preserved stool samples J Clin Microbiol 2001 39 716 719 10.1128/JCM.39.2.716-719.2001 11158133 PMC87802

[B16] Gonin P Louise T Detection and differentiation of Entamoeba histolytica and Entamoeba dispar isolates in clinical samples by PCR and enzyme-linked immunosorbent assay J Clin Microbiol 2003 41 237 241 10.1128/JCM.41.1.237-241.2003 12517854 PMC149615

[B17] Redondo RB Mendez LGM Baer G Entamoeba histolytica and Entamoeba dispar: differentiation by enzyme-linked immunosorbent assay (ELISA) and its clinical correlation in pediatric patients Parasitol Latinoam 2006 6 37 42

[B18] Khairnar K Parija SC A novel nested multiplex polymerase chain reaction (PCR) assay for differential detection of Entameoba histolytica, E. moshkovskii and E. dispar DNA in stool samples BMC Microbiol 2007 7 47 10.1186/1471-2180-7-47 17524135 PMC1888694

[B19] Zeehaida M Wan Nor Amilah WAW Amry AR Hassan S Sarimah A Rahmah N A study on the usefulness of Techlab Entamoeba histolytica II antigen detection ELISA in the diagnosis of amoebic liver abscess (ALA) at Hospital Universiti Sains Malaysia(HUSM), Kelantan, Malaysia Trop Biomed 2008 25 209 216 19287359

[B20] Parija SC Garg A Puspha K Khairnar K Priya T Polymerase chain reaction of diagnosis of intestinal amebiasis in Puducherry Indian J Gastroenterol 2010 29 140 142 10.1007/s12664-010-0033-0 20814775

[B21] Norhayati M Fatmah MS Yusof S Intestinal parasitic infections in man: A review Med J Malaysia 2003 58 2 10 14569755

[B22] Lim YAL Romano N Colin N Chow SC Smith HV Intestinal parasitic infections amongst Orang Asli (indigenous) in Malaysia: Has socioeconomic development alleviated the problem? Trop Biomed 2009 26 110 122 19901897

[B23] Ngui R Saidon I Chow SK Rohela M Lim YAL Prevalence and risks factors of intestinal parasitism in rural and remote West Malaysia PLoS Negl Trop Dis 2011 5 e974 10.1371/journal.pntd.0000974 21390157 PMC3046966

[B24] Nor A Ashley S Albert J Parasitic infection in human communities living on the fringes of the Crocker Range Park Sabah, Malaysia ASEAN Review of Biodiversity and Environmental Conservation (ARBEC) 2003 http://www.arbec.com.my/pdf/art11janmar03.pdf

[B25] Rajeswari B Sinniah B Hussein H Socioeconomic factor associate with intestinal parasites among children living in Gombak, Malaysia Asia Pac J Publ Health 1994 7 21 25 10.1177/101053959400700104 8074940

[B26] Hakim SL Gan CC Malkit K Azian MN Chong CK Shaari N Zainuddin W Chin CN Sara Y Lye MS Parasitic infections among Orang Asli (aborigine) in the Cameron Highlands, Malaysia Southeast Asian J Trop Med Publ 2007 38 415 419 17877212

[B27] Que X Reed SL Nucleotide sequence of a small subunit ribosomal RNA (16S like rRNA) gene from Entamoeba histolytica: differentiation of pathogenic from non pathogenic isolates Nucleic Acids Res 1991 19 5438 10.1093/nar/19.19.5438 1923831 PMC328914

[B28] Noor Azian MY San YM Gan CC Yusri MY Nurulsyamzawaty Y Zuhaizam AH Maslawaty MN Norparina I Vythilingam I Prevalence of intestinal protozoa in an aborigine community in Pahang, Malaysia Trop Biomed 2006 24 55 62 17568378

[B29] Sukprasert S Rattaprasert P Hamzah Z Shipin OV Chavalitshewinkoon-Petmitr P PCR detection of Entamoeba spp from surface and waste water samples using genus-specific primers Southeast Asian J Trop Med Publ 2008 39 6 9

[B30] Petri JWA Haque R Lyerly D Vines R Estimating the impact of amoebiasis on health Parasitol Today 2000 16 320 321 10.1016/S0169-4758(00)01730-0 10900473

[B31] Visser LG Verweij JJ Van Esbroeck M Edeling WM Clerinx J Polderman AM Diagnostic methods for differentiation of Entamoeba histolytica and Entamoeba dispar in carriers: performance and clinical implications in a non-endemic setting Int J Med Microbiol 2006 296 397 403 10.1016/j.ijmm.2006.03.001 16753339

[B32] Haque R Ali IKM Clark CG Petri JWA A case report of Entamoeba moshkovskii infection in a Bangladeshi child Parasitol Int 1998 47 201 202

[B33] Farhana F Jamaiah I Rohela M Abdul-Aziz NM Nissapatorn V A ten year (1999–2008) retrospective study of amoebiasis in University Malaya Medical Centre (UMMC), Kuala Lumpur, Malaysia Trop Biomed 2009 26 262 266 20237439

[B34] Kebede A Verweij JJ Endeshaw T Messele T Tasew G Petros B Polderman AM The use of real-time PCR to identify Entamoeba histolytica and E. dispar infections in prisoners and primary-school children in Ethiopia Ann Trop Med Parasitol 2004 98 43 48 10.1179/000349804225003082 15000730

